# Longitudinal comparison of psychological outcomes of professional content moderators engaged in resilience training program

**DOI:** 10.3389/fpsyt.2025.1666543

**Published:** 2025-12-04

**Authors:** Marlyn Thomas Savio, Aruna Balammal, John Caesar de Villa, Jolguer Perez, Xieyining Huang, Rachel Lutz Guevara

**Affiliations:** Wellness & Resiliency Division of Research, TaskUs Inc., New Braunfels, TX, United States

**Keywords:** content moderation, psychological safety, resilience, secondary trauma, wellness intervention

## Abstract

**Introduction:**

Content moderators safeguard the ever-growing online realm by reviewing and filtering out harms, yet are themselves subject to the risk of psychological concerns.

**Method:**

This study explored the psychological outcomes of content moderators (N = 311) who were offered a resilience training program called The TaskUs Method 2.0 from baseline to 18 months. Participants completed psychometric screeners for resilience, burnout, secondary traumatic stress, compassion satisfaction, and perceived stress. For the analyses, three timepoints were identified to follow participants across the study period based on their tenure–T0 (0–3 months tenure), T1 (5–10 months), and T2 (11–16 months tenure).

**Results:**

Findings from the entire sample (regardless of whether participants had data for all three timepoints) revealed that secondary traumatic stress and perceived stress did not differ significantly between timepoints. However, burnout, compassion satisfaction and resilience showed small yet significant decline between T0 and T2. With a reduced sample of only those having data for all three timepoints, similar trends remained although the rate of change from T0 to T1 did not differ significantly from that of T1 to T2 for any of the variables.

**Discussion:**

Results indicated that participants in the program showed no significant increases in secondary traumatic stress, the main target of the program. Across the different psychometrics, effect sizes were small suggesting that content moderators using the program were not at significant risk for increased psychological distress from content moderation during the study duration. These findings provide a valuable foundation for further refining interventions aimed at content moderators. Future studies may consider experimental and multifactorial designs to delve into therapeutic mechanisms and different types of psychological outcomes.

## 1 Introduction

In an age characterized by rapid technological epiphanies, the continued safety of digital spaces is critically dependent on content moderators. They review user generated material on social media, streaming and networking platforms in varying formats for alignment with usage policies, and take actions on violative material. The World Economic Forum lamented the ease the internet offers for the widespread movement of harmful content (1). While not enough formal data is accessible on the current global volume of violative content, a recent report from the UK revealed a 31% increase in harmful content in 2023 when compared to 2022 (2). Moderators, thus, face more recurrent risks of engagement with traumatic content (3). Repeated exposure can trigger mental health concerns, and calls for efforts to protect moderators from the negative impacts of content on their well-being (4). This study examines the longitudinal trends of self-reported psychological outcomes of content moderators while using a tailored psychological health intervention by tracking the well-being outcomes of participating content moderators across 18 months.

Historically, moderation work was managed on a small scale by existing employees of a given platform. Over the years, the sheer volume and diversity of user generated content has required content moderation to be set up as a full-fledged specialized department. While the job role and expectations for moderators have become more refined and systematized, attention to the human consequences of such work has not been on par, and even fewer efforts have been directed to alleviate possible occupational impact (5, 6). Prolonged interaction with harmful content while moderating potentiates the development of trauma-related symptoms and disorders. In frontline professions such as content moderation, secondary traumatic stress, burnout and compassion fatigue are very relevant and necessitate tailored mitigation strategies (4).

Secondary traumatic stress is commonly experienced by helping professionals (helpers) as they continually witness and work with victims, survivors, and records of perpetrations. Even while helpers such as content moderators may not personally encounter the traumatic incident, the nature and responsibility of helping work that involves empathizing and safeguarding others (i.e., platforms users in content moderation contexts) creates trying scenarios that can potentially trigger traumatic responses in moderators (7). Long-term helping demands emotional labor without sacrificing consistent work performance, which altogether can result in burnout (characterized by exhaustion, hopelessness, and maladaptive coping) as well as compassion fatigue (reduced ability to empathize or help) (8). In a study with news media staff, it was found that empathy or compassion expressed in one’s work moderated the impact of negative emotions on rumination tendencies which in turn mediated burnout (9). There thus appears to be a psychological pathway between traumatic contact on the job and the possible development of distress.

The elucidation of the empathy mechanism, nonetheless, offers hope to disrupt the progression from exposure to psychological injury, by training helpers in healthy coping techniques. The World Health Organization (10) acknowledged the existence of “elevated psychosocial risks” for helpers, and recommends employers to offer preventative psychoeducative training alongside other risk reduction provisions. In recent years, wellness benefits have become more common in workplaces (11). These, however, are often reactive care set-ups that place the onus on employees to reach out when in detectable distress (which may be too late). Singh et al. (12) argued for anticipatory/preventative mental health care in communities to avoid aggravations of undetected subclinical conditions, and to reduce the burden of self-diagnosis. For full-time content moderators, this is even truer given the known psychological risks in their occupation. To our knowledge, very limited scientific literature exists on wellness interventions for content moderators. Most reports have been centered on content with the goal to reduce its shock value and in turn minimize the emotional impact on moderators. These have yielded mixed results. On one hand some studies (13–15), evaluating technological interventions such as image modification through blurring and filters, found positive effects like reduced negative feelings while retaining operational efficiency. Contrastingly, in another study (16) that tried to induce positive affect (e.g., introducing cute and awe-inspiring images/videos) during work breaks, experienced moderators (with 2+ years of moderation experience) reported higher perceived stress. The authors proposed that compassion fatigue (stemming from moderators’ prolonged exposure to content) had reversed the effect of surface-level positive stimuli, thus requiring interventions that focus on the individual and address deeper psychological concerns. While content-focused interventions can support the reduction of impact, these (in and by themselves) cannot holistically protect moderators. They address merely a part of the problem (content), and do not leverage the human capacity to cope with distress.

The only comprehensive preventative intervention for content moderators documented in empirical literature to date is Steiger et al.’s (17) resilience training program called the TaskUs Method. The cyclical intervention, involving positive psychological education and theme-based activities to promote emotional regulation and adaptive coping was observed to prevent burnout and secondary traumatic stress from worsening between moderators’ 2-week and 3-month tenure timepoints. Furthermore, higher resilience predicted lower burnout and secondary traumatic stress. The study highlighted the benefits of a periodic intervention that fosters resilience in moderators to help them withstand psychosocial risks. However, two limitations are notable: the study was conducted during the stressful COVID-19 pandemic, and its long-term impact beyond the initial 3-month habituation phase for moderators remains unclear. Given that the demands of content moderation are continually evolving with the advance of digital content and newer technologies (e.g., GenAI, virtual reality), interventions must be designed to instill skills that help moderators cope with new risks and changing dynamics of their job.

This study builds on Steiger et al.’s (17) intervention report by exploring the second iteration of the program. The modules in the TaskUs Method 2.0 version were designed to be tailored, trauma-responsive and stress-inoculating in the context of content moderation work. Practical everyday skills (e.g., tricks for nervous system regulation) rather than abstract mechanisms of coping (e.g., optimistic worldview) were prioritized. The revised approach aimed to retain the central focus on resilience building against trauma for moderators while being more experiential than theoretical. This article reports the well-being outcomes of full-time content moderators participating in the TaskUs Method 2.0 program by following them up for 18 months.

## 2 Method

### 2.1 Participants

The sample comprised 311 full-time content moderators–employed by TaskUs, a business process outsourcing (BPO) company–whose primary job responsibility included reviewing material classified as highly graphic or egregious. Nonetheless, the moderators were exposed to content that vary in format (text, images, or videos) and type of harm (e.g., bullying, child abuse, violence, hate, or misinformation). All content moderators at TaskUs are hired with the help of a psychometric pre-hire screener (18) designed to assess a candidate’s cognitive and psychological traits to both predict success in the role and mitigate the risk of adverse outcomes (e.g., secondary traumatic stress). The hiring process also includes interviews and discussions to ensure role transparency as well as candidates’ acknowledgement and informed willingness to work in content moderation.

Participants were located in Southeast Asia (77%), India (20%), Latin and North Americas (2%), and Europe (1%). This regional distribution reflected the typical content moderator distribution in BPOs, with a majority located in Asia and smaller proportions in the west. As regards age distribution, 38.6% of the participants were of 25–30 years, 32.2% fell into 18–24 years, 13.8% were of 31–35 years, 8.4% fell into 36–40 years, 3.5% were of 41–50 years, and 2.9% chose not to disclose.

### 2.2 Measures

#### 2.2.1 Professional quality of life

ProQOL, developed by Stamm (19), includes 30 items that enquire about one’s feelings with regard to helping work during the last 30 days. It has three sub-scales with 10 items each on a 5-point likert scale of frequency that evaluate burnout (e.g., “I feel overwhelmed because my work load seems endless.”), secondary traumatic stress (e.g., “Because of my helping, I have felt “on edge” about various things.”), and compassion satisfaction (“I get satisfaction from being able to help people.”). For each subscale the score ranges between 10 and 50, and interpreted as follows—between 10 and 22 represents low range, between 23 and 41 signifies average/moderate range, and above 41 indicates high range. The internal consistency of ProQOL subscales, gauged by Cronbach’s alpha, in the present sample ranged at the three timepoints (T0, T1, T2) between 0.87 and 0.89 (secondary traumatic stress), 0.74 and 0.81 (burnout), and 0.87 and 0.90 (compassion satisfaction).

#### 2.2.2 Perceived stress scale

PSS is a 10-item scale from Cohen et al. (20), evaluating the extent to which one’s life situation had been stressful during the past month on a 5-point likert scale of frequency. Items are both positively and negatively phrased to cover different stressful and coping experiences (e.g., “In the last month, how often have you felt confident about your ability to handle your personal problems?”, “In the last month, how often have you been angered because of things that were outside of your control?”). The total score ranges between 0 and 40, with 0–13 suggesting low stress, 14–26 indicating moderate stress, and above 27 representing high stress. PSS had internal consistency (Cronbach’s alpha) of 0.87 at all timepoints in this study.

#### 2.2.3 Connor-Davidson resilience scale 10

CD-RISC 10 assesses different aspects of an individual’s resilience capacity or their ability to recover from stressful events (e.g., “I am not easily discouraged by failure.”). The 10-item version with a 5-point likert scale was adapted by Campbell-Sills & Stein (21) from the original 25-item scale by Connor & Davidson (22). The total score falls between 0 and 40, such that 0–29 is considered low range, 30–36 is suggestive of moderate/intermediate range, and 37–40 is seen as high range (23). In the present sample, the internal consistency (Cronbach’s alpha) of CD-RISC 10 was in the range of 0.87 and 0.90 for the different timepoints.

### 2.3 Study procedure and program

The study involved secondary analyses of data obtained from psychometric surveys conducted by the authors at 6-month intervals between May 2022 and November 2023 with employees in the organization. During this period the resilience program was administered to participants by licensed mental health professionals and trained coaches (not researchers). Employees were offered wellness time (paid for by employer) for the wellness program during their work shift, resulting in an observed attendance of 80-90%. The researchers were involved only in conducting the survey, and they had no oversight or management of the program delivery team. The surveys were administered for four rounds at 6-month intervals (May 2022, November 2022, May 2023, and November 2023).

The resilience program was designed by licensed mental health professionals as an evidence-based holistic, global psychological health and safety framework for full-time content moderators. The offerings were based on neuroscience principles and psychology practices (24–28). The goal of the sessions was to foster neuroplasticity and improve moderators’ stress tolerance as well as healthy coping techniques (29). Theoretically, the program was founded on brain-based and mind-body interventions (26, 28) namely, Eye-Movement Desensitization and Reprocessing (30, 31), Somatic Experiencing (32–34), and Rapid Resolution Therapy (35, 36), coupled with cognitive modalities to include Cognitive Processing Therapy (27, 37–39) and Dialectical Behavioral Therapy (25, 40, 41).

The underlying structure of these skill-building sessions was experiential adaptability, wherein attendees experience, in real-time, activities that cause positive changes in the brain for optimal functioning. This approach minimizes the “teaching” aspect and maximizes experience. This focus on direct experience aimed to ensure deep, lasting integration of skills. Sessions were periodically offered to moderators during wellness time in their shift to facilitate mastery and reinforcement of adaptive skills. The skill sessions were arranged in a build-over-the-weeks progression with specific themes across the months: cognitive control and flexibility, benefits of music, multiplicity of emotional selves, benefits of mobility, optimizing teamwork, visuospatial gaming, creating safety, harnessing imagination, nervous system regulation, and nurturing inner speech. Techniques used during these sessions to increase hands-on participation included demonstrations, analogies, role plays, goal setting, discussions, reflections, purposeful games, and creative expressions. Research suggests that these activities and topics support resiliency and psychological functioning, especially in those exposed to graphic materials or who have experienced a traumatic event (42–44).

The themes remained consistent across the regions where participants hailed from. Nonetheless, local facilitators (i.e., coaches and licensed professionals) would adapt the material or exercises to suit community expectations and cultural norms.

### 2.4 Ethical considerations

Survey data were gathered purely for research purposes in the organization, and no individual-level data was shared anywhere or with anyone outside the research team. The research team was independent of the operations department as well as the resilience intervention delivery team, with no direct oversight or management of participants’ work, thereby mitigating conflict of interest.

Participation was purely voluntary and confidential. Written informed consent was obtained from all participants prior to their participation in the surveys. This was in accordance with ethical standards for the protection of human participants. Specifically, participants were presented with a comprehensive study information sheet that detailed the purpose and process of the survey, and explained the voluntary nature of participation alongside the right to withdraw at any time without penalty. Participants were assured that their responses had no bearing on employment and performance KPIs. Furthermore, the information sheet described the measures taken to ensure data confidentiality (including de-identification and aggregation for analysis), potential minimal anticipated harms or discomforts (with contact information for seeking help), and the secure procedures for data management throughout the study duration.

Given that the study involved secondary analysis of deidentified data, the study was given the exemption from IRB review in writing by the ethics review board at TaskUs Inc., chaired by a mental health researcher from an external research and academic institution. No adverse outcomes were reported during the study period.

### 2.5 Data analyses

**Figure 1:**
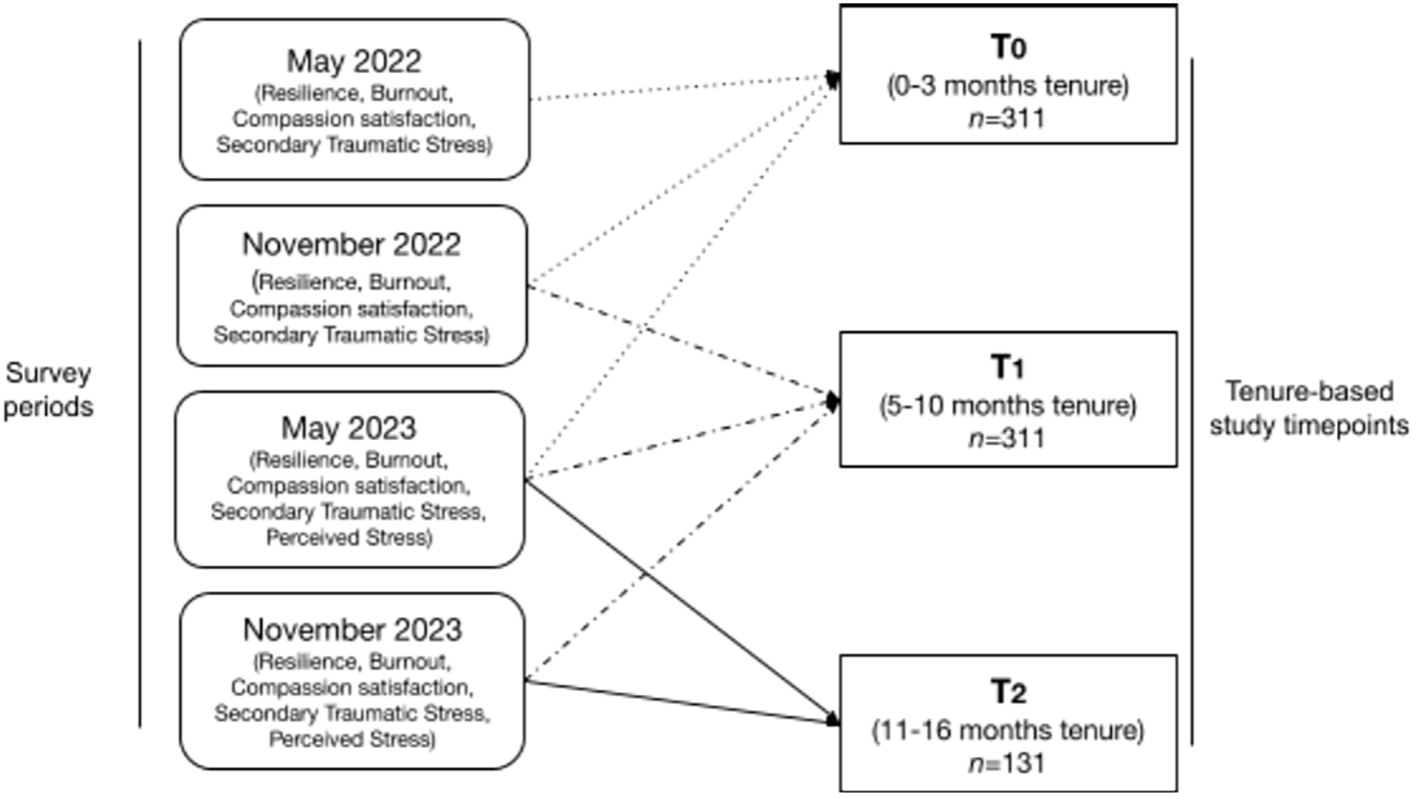
Study timeline and sample composition.

Participants were grouped into three time points (T0, T1, and T2) based on their organizational tenure. Each time point thus represents the sample defined by their tenure even if they had taken the survey in different calendar quarters. **Figure 1** illustrates the sample composition from across the survey rounds between May 2022 and November 2023. Timepoints corresponded to how long they had been with the company at the time of the quarterly survey, thus representing tenure time rather than specific dates. Timepoint 0 (T0) contained 311 moderators who had worked 0–3 months at the time of the survey (M = 2.15, SD = 0.86). For Timepoint 1 (T1), moderators with a tenure of 5–10 months were included, i.e., all 311 participants qualified (M = 7.81, SD = 1.11). Timepoint 2 (T2), however, contained fewer individuals (N = 131) than the original 311 participants as it required participants who had been working for 11–16 months within the survey window. Individuals without a T2 could have left the company prior to reaching the third timepoint or simply started working later within the previous survey window and thus had not yet completed a third survey. It is also likely that they may have remained employed but chose not to respond to the survey given the voluntary nature of participation. The reason for the participants’ dropout (i.e., whether due to non-participation or exit from company) cannot be confirmed with available data, meaning it could be for different reasons not tracked here.

Perceived stress was added to the survey later so participants will only have data on that variable for surveys completed in the two most recent surveys (May 2023 and November 2023), meaning that no participants have data at all three timepoints for perceived stress, and which timepoints are available is based on what year/quarter survey corresponded to each timepoint for that individual. That is, only participants who started after March 2023 would have data for perceived stress at T0 (having their first quarterly survey in May or November 2023). There were 110 individuals who had data at T0, 208 at T1, and 131 at T2. Further, there were 110 individuals who had data at both T0 and T1, and 69 who had data at both T1 and T2.

Paired t-tests were conducted to compare psychological outcomes across timepoints to examine whether the change across time is significantly different from zero. Follow-up analyses were conducted to compare the amount of change observed between T0 and T1 compared to T1 to T2. In order to be able to directly compare the changes, only participants with all three timepoints were used in the follow up analysis. Since no participants had perceived stress data at all three timepoints, this outcome was not examined in the follow-up analyses. First, paired t-tests were conducted in the same way as on the full sample but this time with only a sample of individuals who had all three timepoints. Next, in order to directly compare whether the change was significantly different between timepoints, the difference score from T0 to T1 was compared to the difference between T1 and T2 using paired t-tests.

## 3 Results

**Figure 2:**
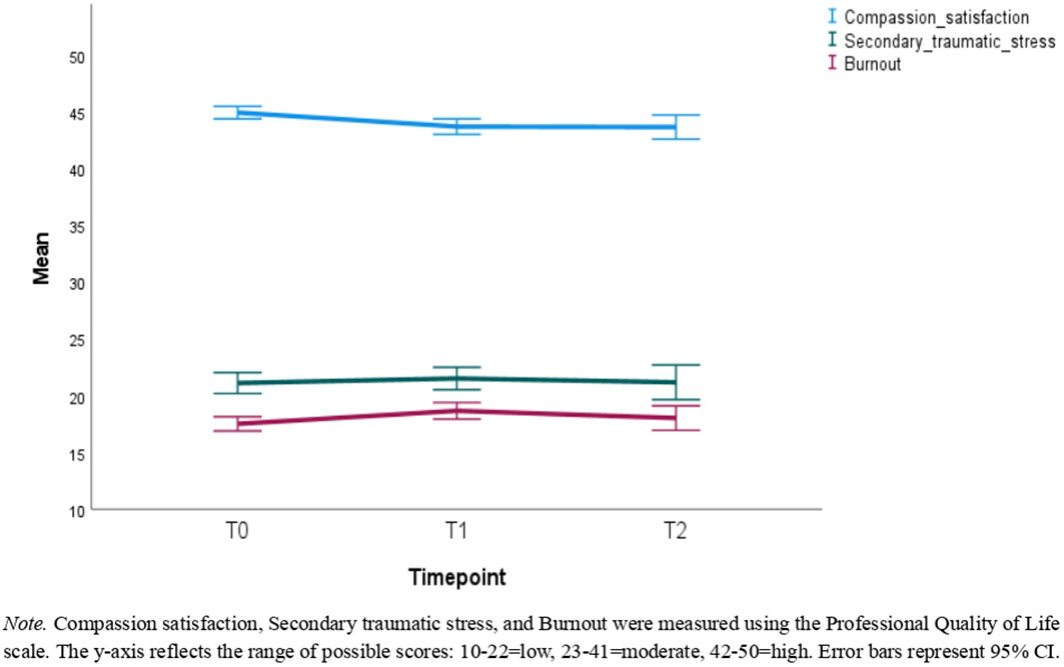
Mean scores of the full sample for compassion satisfaction, secondary traumatic stress, and burnout across the study timepoints.

**Figure 3:**
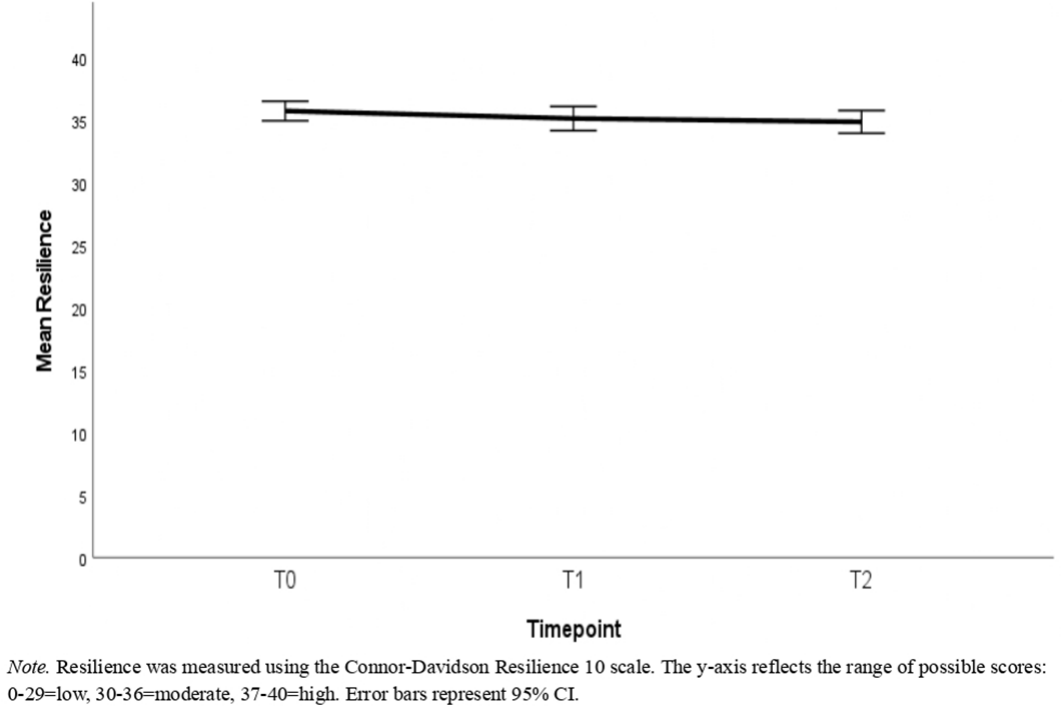
Mean scores of the full sample for resilience across the study timepoints.

**Figure 4:**
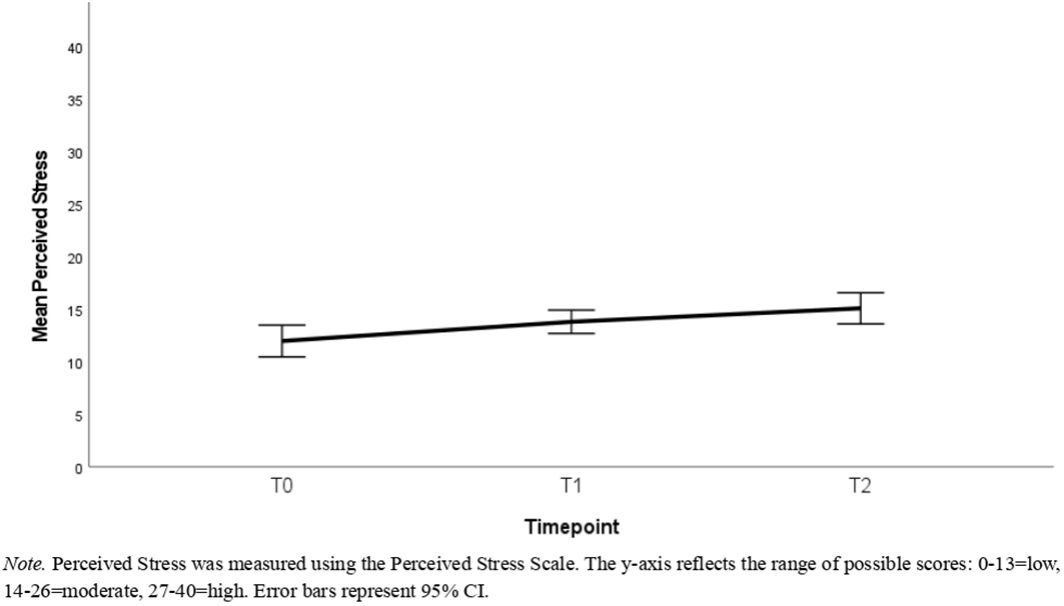
Mean scores of the full sample for perceived stress across the study timepoints.

**Figure 5:**
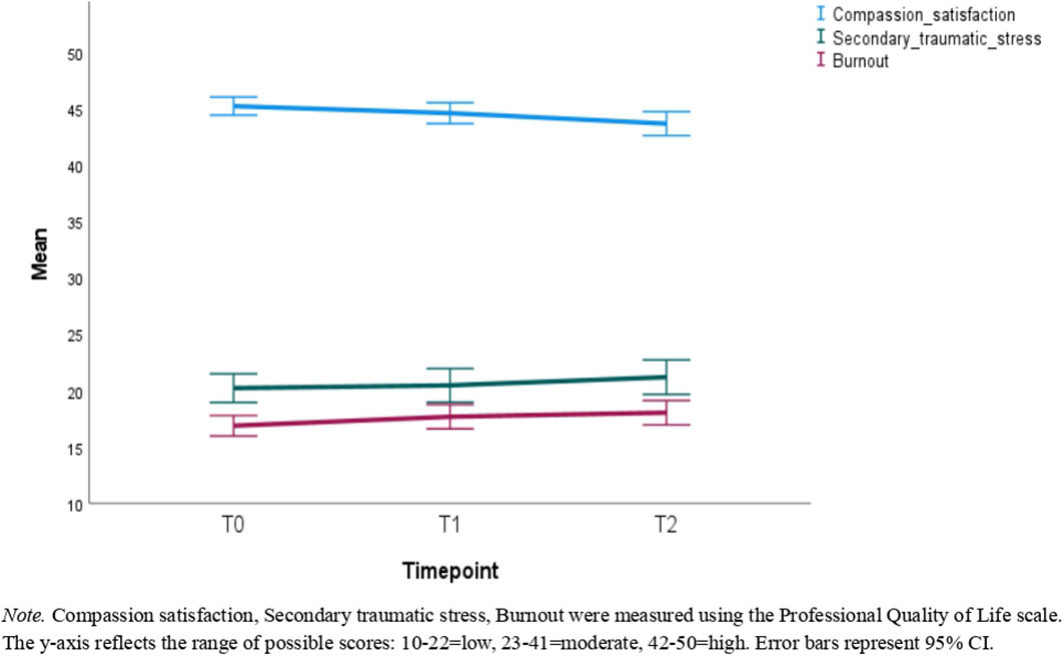
Mean scores of the reduced sample for compassion satisfaction, secondary traumatic stress, and burnout across the study timepoints.

**Figure 6:**
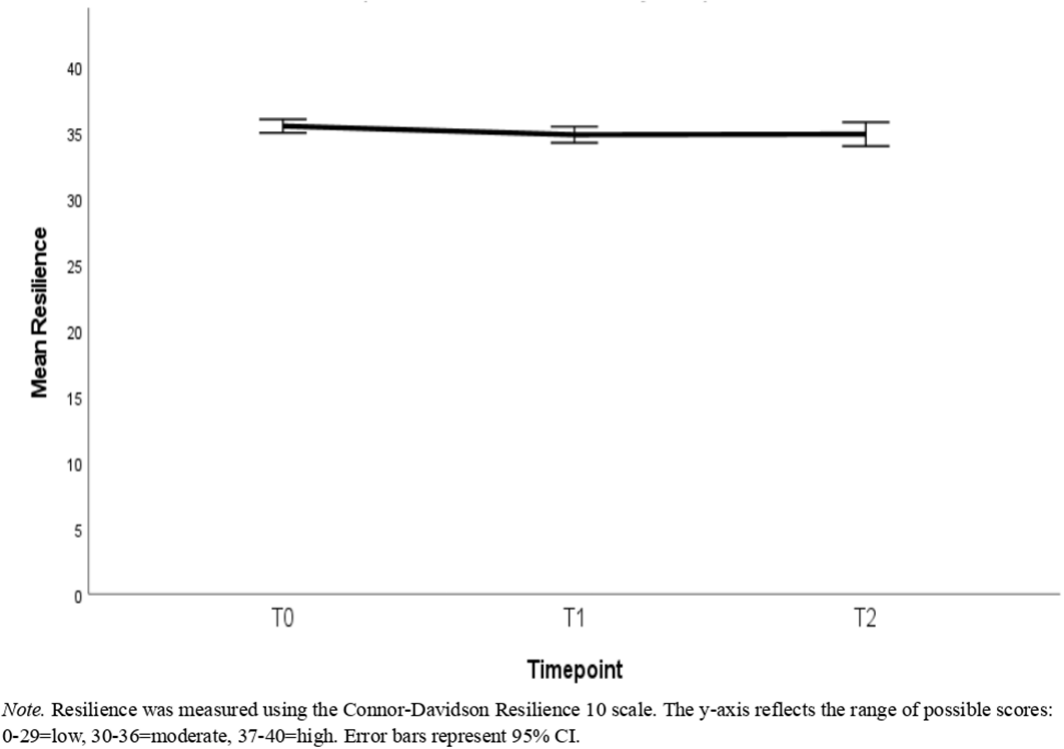
Mean scores of the reduced sample for resilience across the study timepoints.

Descriptive statistics can be found in **Tables 1**–**3**. **Table 1** reports the number of participants with available data, means, and standard deviations for each timepoint in the full sample. **Table 2** reports this information for the reduced sample of participants that had data available at all timepoints. **Table 3** presents the correlations among these variables with the correlations in the full sample being represented in the lower triangle, whereas the upper triangle contains correlations from the reduced sample. **Figures 2**–**4** depict the mean scores for compassion satisfaction, secondary traumatic stress, burnout, resilience and perceived stress in the full sample across timepoints. **Figures 5**, **6** illustrate the mean scores for compassion satisfaction, secondary traumatic stress, burnout, and resilience in the reduced sample across timepoints.

**Table 1:** Descriptive statistics across timepoints for the full sample.

	T0			T1			T2		
	n	Mean	SD	n	Mean	SD	n	Mean	SD
Months of employment	311	2.15	0.86	311	7.81	1.11	131	13.58	0.94
Secondary Traumatic Stress	311	21.10	8.28	311	21.50	8.88	131	21.16	8.81
Perceived Stress	110	11.95	8.01	208	13.78	8.12	131	15.07	8.58
Resilience	311	35.49	4.62	311	34.83	5.52	131	34.87	5.24
Burnout	311	17.50	5.59	311	18.65	6.55	131	18.02	6.25
Compassion Satisfaction	311	44.90	4.96	311	43.67	6.09	131	43.63	6.15

**Table 2:** Descriptive statistics across timepoints for the reduced sample.

	T0			T1			T2		
	n	Mean	SD	n	Mean	SD	n	Mean	SD
Months of employment	131	2.26	0.84	131	7.98	1	131	13.58	0.94
Secondary Traumatic Stress	131	20.19	7.43	131	20.44	8.63	131	21.16	8.81
Perceived Stress	0	–	–	69	14.48	8.48	131	15.07	8.58
Resilience	131	35.74	4.54	131	35.14	5.59	131	34.87	5.24
Burnout	131	16.85	5.25	131	17.66	6.21	131	18.02	6.25
Compassion Satisfaction	131	45.18	4.65	131	44.56	5.34	131	43.63	6.15

**Table 3:** Correlations: lower triangle contains correlations from the full sample, upper triangle contains correlations from the reduced sample.

	1	2	3	4	5	6	7	8	9	10	11	12	13	14	15	16	17	18
1. T0 tenure	1	-0.04	0.1	NA	0.07	-0.03	0.67	0	0	0.06	0.09	0.02	0.8	0	-0.02	-0.03	0.07	0.04
2. T0 Burnout	0.07	1	-0.51	NA	-0.38	0.67	-0.14	0.64	-0.44	0.47	-0.4	0.48	-0.1	0.5	-0.42	0.43	-0.34	0.31
3. T0 Compassion Satisfaction	-0.05	-0.58	1	NA	0.58	-0.02	0.12	-0.4	0.49	-0.3	0.31	-0.2	0.07	-0.4	0.41	-0.35	0.38	-0.17
4. T0 Perceived Stress	0.2	0.61	-0.53	1	NA	NA	NA	NA	NA	NA	NA	NA	NA	NA	NA	NA	NA	NA
5. T0 Resilience	-0.08	-0.42	0.54	-0.49	1	-0.07	0.06	-0.3	0.39	-0.5	0.52	-0.2	0.03	-0.4	0.35	-0.45	0.56	-0.2
6. T0 Secondary Traumatic Stress	0.09	0.71	-0.2	0.49	-0.15	1	-0.09	0.43	-0.16	0.43	-0.2	0.56	-0.05	0.2	-0.27	0.25	-0.15	0.29
7. T1 tenure	0.75	0.09	-0.13	0.27	-0.15	0.07	1	0	-0.01	0.1	0.02	0.09	0.84	0	-0.06	-0.03	0.01	0.13
8. T1 Burnout	0.04	0.63	-0.5	0.54	-0.43	0.47	0.11	1	-0.64	0.7	-0.5	0.71	0.02	0.8	-0.45	0.57	-0.42	0.59
9. T1 Compassion Satisfaction	-0.07	-0.48	0.53	-0.47	0.45	-0.28	-0.13	-0.7	1	-0.6	0.7	-0.2	-0.04	-0.6	0.64	-0.44	0.58	-0.26
10. T1 Perceived Stress	0.12	0.45	-0.42	0.5	-0.52	0.39	0.2	0.72	-0.58	1	-0.6	0.61	0.18	0.6	-0.41	0.72	-0.41	0.55
11. T1 Resilience	-0.08	-0.36	0.38	-0.45	0.56	-0.19	-0.17	-0.6	0.68	-0.6	1	-0.2	0.02	-0.6	0.58	-0.46	0.73	-0.25
12. T1 Secondary Traumatic Stress	0.08	0.52	-0.31	0.41	-0.29	0.58	0.13	0.74	-0.32	0.65	-0.3	1	0.1	0.5	-0.17	0.43	-0.18	0.71
13. T2 tenure	0.8	-0.1	0.07	NA	0.03	-0.05	0.84	0.02	-0.04	0.18	0.02	0.1	1	0.1	-0.06	-0.02	0.03	0.13
14. T2 Burnout	0.02	0.52	-0.41	NA	-0.37	0.24	0.04	0.75	-0.6	0.57	-0.6	0.5	0.06	1	-0.61	0.69	-0.6	0.69
15. T2 Compassion Satisfaction	-0.02	-0.42	0.41	NA	0.35	-0.27	-0.06	-0.5	0.64	-0.4	0.58	-0.2	-0.06	-0.6	1	-0.44	0.68	-0.16
16. T2 Perceived Stress	-0.03	0.43	-0.35	NA	-0.45	0.25	-0.03	0.57	-0.44	0.72	-0.5	0.43	-0.02	0.7	-0.44	1	-0.55	0.5
17. T2 Resilience	0.07	-0.34	0.38	NA	0.56	-0.15	0.01	-0.4	0.58	-0.4	0.73	-0.2	0.03	-0.6	0.68	-0.55	1	-0.23
18. T2 Secondary Traumatic Stress	0.04	0.31	-0.17	NA	-0.2	0.29	0.13	0.59	-0.26	0.55	-0.3	0.71	0.13	0.7	-0.16	0.5	-0.23	1

This section reports, at the outset, the findings of analyses with the entire sample (N = 311) regardless of the number of timepoints participants had to showcase the overall group’s well-being status. In the next part, the findings of participants with all 3 time-points (n=131) are presented to show the extent of differences in well-being aspects across time.

### 3.1 Full sample analyses

#### 3.1.1 Secondary traumatic stress

Paired t-tests revealed that secondary traumatic stress scores at T1 were not significantly different from that of T0 [t (310) = .895, p=.371, d = .05], and T2 was not significantly different from T1 [t(130) = 1.24, p=.216, d = .11]. Additionally, when comparing T2 to T0, the difference in secondary traumatic stress was not significantly different from zero [t(130) = 1.14, p=.255, d = .10].

#### 3.1.2 Perceived stress

Paired t-tests showed no significant difference in scores between T1 compared with T0 [t(109) = 1.39, p=.169, d = .13] or T2 compared to T1 [t(68) = .32, p=.748, d = .04]. No participants had data available for both T0 and T2, thus this difference was not compared.

#### 3.1.3 Resilience

Results of a paired t-test demonstrated that scores on the resilience scale were significantly lower at T1 than T0 with an average decrease of 0.65 [t(310) = -2.42, p=.016, d = -.14]. However, the difference between T1 and T2 was not significant [t(130)=-.76, p = .447, d = -.07]. Additionally, T2 resilience was significantly lower than T0 with an average decrease of.87 [t(130) = -2.14, p=.034, d = .19].

#### 3.1.4 Burnout

Burnout at T1 was significantly higher than at T0 with an average difference of 1.15 [t(310)=3.82, p <.001, d = .22]. However, the difference in burnout between T1 and T2 was not significant [t(130) = .93, p = .351, d = .08]. The overall change from T0 to T2 was significant such that participants had an average of 1.16 higher burnout scores at T2 than at T0 [t(130) = 2.32, p = .022, d = .20].

#### 3.1.5 Compassion satisfaction

Paired t-tests revealed that compassion satisfaction at T1 was 1.23 lower on average than at T0 [t(310)=-3.99, p<.001, d = -.23] and.93 lower at T2 compared to T1 [t(130)=-2.17, p=.032, d = -.19]. Compassion satisfaction was also 1.55 lower on average at T2 than T0 [t(130)=-2.97, p=.004, d = -.26].

### 3.2 Reduced sample analyses for follow-up comparisons on the amount of change over time

#### 3.2.1 Secondary traumatic stress

When reduced to only the participants with all three timepoints, no significant difference was observed between T0 and T1 [t(130)=.37, p=.711, d = .03], T1 and T2 [t(130) = 1.24, p=.216, d = .11], or T0 and T2 [t(130) = 1.14, p=.255, d = .10]. The amount of change between T0 and T1 was not significantly different than that of T1 to T2 [t(130)=.528, p=.598, d = .05].

#### 3.2.2 Resilience

When reduced to only the sample with all three timepoints, the magnitude of the difference between T0 and T1 resilience decreased only slightly with an average difference of.60 (compared to.65 in the full sample), but this difference was no longer statistically reliably different from zero [t(130) = -1.37, p=.173, d = .12]. As discussed above, the change from T1 to T2 with an average difference of.27 was not statistically reliable [t(130)=-.76, p = .447, d = -.07] but the 95% confidence interval (.07, 1.67) did not include 0 for the difference between T0 and T2 which showed an average decrease of.87 [t(130) = -2.14, p=.034, d = .19]. The magnitude of the change from T0 to T1 was not significantly different from that of T1 to T2 [t(130)=.49, p=.625, d = .04].

#### 3.2.3 Burnout

Similarly, the difference in burnout from T0 to T1 was no longer statistically reliable with an average increase of.80 in the reduced sample [t(130)=1.85, p=.066, d = .16]. When comparing this change to that of T1 to T2 reported above (non-significant average of.36, d = .08) this difference was not statistically significant [t(130)=.68, p=.495, d = .06].

#### 3.2.4 Compassion satisfaction

Compassion satisfaction also showed a smaller difference from T0 to T1 when reduced to only participants with all timepoints with the average difference in this reduced sample being.62 (compared to 1.23 in the full sample) and this difference was no longer significant [t(130)=1.40, p=.164, d = .12]. Comparing this decrease to that of T1 to T2 (reported above) which was.93 on average, results revealed that the amount of change observed by an individual in T0 to T1 was not significantly different from their change from T1 to T2 [t(130)=.45, p=.654, d = .04].

## 4 Discussion

The aim of this study was to examine the well-being outcomes of content moderators who received the TaskUs Method 2.0 resilience training intervention. Overall, the investigation revealed that participants in the program maintained stable psychometric scores across most measures. Participants did not show significant worsening in secondary traumatic stress and perceived stress between baseline and 18 months. Outcomes such as burnout and resilience differed significantly yet with small effect size. The findings indicate that when using the program, content moderators in this study were not at risk for large declines in well-being outcomes. The results are discussed and contextualized in detail below.

The primary aim of the intervention was to promote resilience among moderators. Findings indicate that the current sample maintained moderate resilience across the study period. While there is limited comparative data on resilience from the same population, studies based on adjacent frontline professions are helpful within reasonable limits. The mean resilience score in the current sample ranging between 34 and 35 (moderate resilience as per norms) is higher than those reported for ambulance personnel (28.9) and U.S. Army combat troops (28.1) when using CD-RISC (23). Given the absence of a true control group in the current study, the past literature offers context to understand the level of resilience in frontline workers.

Secondary traumatic stress is arguably the most prominent psychological health concern in content moderation work given the unique firsthand exposure of moderators to unfiltered harmful digital content. Results showed that moderators using the current program maintained status quo in secondary traumatic stress, similar to resilience. This is a critical finding in this study underlining the protective effect of the intervention program against traumagenic risks. It sheds light on the possibility that moderators can perform their duties without suffering additional psychological damage for a considerable duration of time. Despite being exposed to potentially disturbing material as part of the job, the current sample of content moderators maintained a consistent level of secondary trauma, likely due to the support and resources provided by the intervention.

In the case of burnout, Steiger et al’s (4) participants showed an analogous trend of no significant increase. In the present study however, burnout in the reduced sample followed a small significant increase between T0 and T2. Nonetheless, the follow-up timeframe in the two studies (3 vs. 18 months) is not comparable. In fact, burnout in this investigation too did not change significantly between T0 and T1 (6 month follow-up). Therefore, the current findings strengthen the case that on average, moderators in the program experience stable levels of burnout at least up to 6 months of participation.

In a similar vein, perceived stress remained unchanged between consecutive time-points (T0 to T1, T1 to T2). Given that content moderation is a demanding, repetitive, and volatile occupation, feeling overwhelmed and frustrated is not uncommon. The finding hints that program participants experience stable stress levels, indicating their ability to cope with and control their everyday stressors. Perceived stress in this role arises from general work demands (e.g., workload, job demands, personal factors) coupled with the exposure to disturbing content (45). While the intervention may have addressed all these factors, individual differences in coping, pre-existing stress, and personal life events likely contributed to the stability of stress levels observed. Nonetheless, as seen with secondary traumatic stress, the intervention appears to prevent significant increase in perceived stress. Compassion satisfaction decreased significantly between T0 and T2 as well as T1 and T2, and resilience significantly decreased between T0 and T2, but the effect sizes were small. These point out the need to revisit the program’s capabilities post the T1 mark. Gradual diminution of resilience and compassion satisfaction might indicate the influence of stressors outside the scope of intervention. Although observed declines were small on average, additional interventional resources and support in these areas may help to eliminate these declines further.

Notably, at all time points, a considerable portion of participants maintained scores within clinically healthy ranges across all constructs (i.e., low burnout, low secondary traumatic stress, high compassion satisfaction, moderate resilience, low to moderate stress). This is consistent with the potential benefit of the intervention, as opposed to prior literature (4, 46), which consistently emphasized the long-term negative psychological impacts of moderation work. In comparison with previous studies on content moderators wherein psychological distress was at clinically moderate to high levels (e.g., 47, 48), in the current sample, lower scores of secondary traumatic stress and burnout for instance across time were observed. Consistency in the face of ongoing exposure to potentially traumatizing content is a noteworthy finding, which suggests that the intervention may contribute to the stability of psychological well-being in this sample. Therefore, this research adds valuable knowledge to a nascent frontline field and helps address the gap in understanding how to support the vulnerable content moderation workforce.

## 5 Limitations and future directions

The primary shortcoming of this design is the lack of randomization and experimental control (standard for causal inference) which restrains the generalizability of results and precludes deductions of direct program impact. Yet, it must be reiterated that the ethics of care (given the established risks in content moderation) in the organization where moderators were working require offering wellness support to moderators. Randomly assigning moderators to a control group without intervention would be no better than withholding a necessary safety measure from individuals known to be at risk. Specifically, this would violate the ethical principle of beneficence, which compels researchers to act in the best interests of participants and to maximize benefits while minimizing potential harm. Furthermore, it represents inequitable treatment. Denying a subset of employees access to this intervention for the sole purpose of empirical comparison in an RCT would expose them to exacerbated harm and would be seemingly unjust and discriminatory from a labor welfare standpoint. Given this scenario we have referenced in the paragraphs above the available evidence from former literature which have documented moderators developing psychological distress and worsening across time; this served as a proxy control against which we have contextualized the outcomes of the current sample. The lack of an internal comparator or control group (due to the ethics of care) prevents us from proposing definitive causal conclusions regarding the intervention.

Alternative methodologies that do not involve withholding a potentially beneficial intervention must be considered, a constraint upon which the present study is designed. For example, studies may explore the possibility of comparing interventions to determine which program is more efficacious and elucidate the mechanisms of interventional effects. Specifically, designs that alter the parts and dosage of the intervention that moderators receive can help us better understand which parts of the intervention are most effective.

Second, the focus was exclusively on self-reported psychological outcomes within a single organization which introduces various reporting and desirability biases. It is worth reiterating nevertheless that the investigators in the study are fully trained in research ethics and executed the present study in compliance with all ethical and labor regulations. They also did not have direct visibility or influence on the operations of the teams from which participants were sourced.

Another consideration is the nature of timepoints. The data were normalized to group the participants based on their tenure at each timepoint, which may not reflect the most reliable trends. The effects of seasonality on work and well-being cannot be ruled out as each study timepoint included data from up to two different times of the year. Additionally, such longitudinal data may have also been affected by self-selection or survival bias (i.e., contains those who did not leave the study and the company). It cannot be confirmed why the sample declined across timepoints (i.e., whether participants exited from the company or study), meaning that the dropout from the study may be considered random at best. Classifying timepoints by participants’ tenure rather than calendar time helped capture a reasonable number of participants at the three timepoints as their experience and exposure in moderation grew. However, this limited the control over other factors such as harm type and sustained sample size.

Grouping participants by the severity of content they work with was also not feasible. For instance, even in dedicated workflows, participants reported the influx of all types of content (e.g., teams working with largely egregious content saw benign or ambiguous content, while teams that work with less severe content encountered outright harmful content periodically). Nevertheless, the training for the job and the current intervention prepared moderators for all types of exposure regardless of the type of their workflow, which helped the study assume reasonable homogeneity in the sample. Prospective studies may consider the inclusion of objective metrics (mental health-related absenteeism and attrition, differentiating between participants leaving the company and those with non-response, controlling different types of harm and risk exposure) and observation reports (e.g., from line managers) alongside a more diverse sample from different organizations to balance the limitations of self-report scales and to increase the representativeness of results.

Collaborative research is the most promising direction ahead to address the demerits discussed and robustly evaluate the efficacy of relevant preventative wellness programs for content moderators. This will allow for sampling moderators from different companies in a randomized controlled design, and produce widely applicable insights regarding interventions for the Trust & Safety frontline.

## 6 Conclusion

Content moderators who were involved in the TaskUs Method 2.0 resilience training program overall demonstrated minimal clinical worsening of psychometric scores between baseline and 18 months of participation. Secondary traumatic stress and perceived stress had the most favorable trend with non-significant changes across time. Compassion satisfaction, resilience, and burnout showed small yet significant decline during the study period. Altogether, the study reinforces the existence of psychological burden on content moderators, and pertinently underlines the importance of preventative and evidence-based interventions for psychological health sustenance. The results of this study additionally warrant further investigation of the TaskUs Method 2.0 resilience training program to gauge its efficacy.

## Data Availability

The raw data supporting the conclusions of this article will be made available by the authors, without undue reservation.
